# Quantitative prediction of radiographic progression in patients with axial spondyloarthritis using neural network model in a real-world setting

**DOI:** 10.1186/s13075-023-03050-6

**Published:** 2023-04-20

**Authors:** In-Woon Baek, Seung Min Jung, Yune-Jung Park, Kyung-Su Park, Ki-Jo Kim

**Affiliations:** 1 Division of Rheumatology, Department of Internal Medicine, Ewha Womans University College of Medicine, Seoul, Republic of Korea; 2 Division of Rheumatology, Department of Internal Medicine, St. Vincent’s Hospital, College of Medicine, The Catholic University of Korea, 93 Jungbu-Daero, Paldal-Gu, Suwon, Gyeonggi-Do 16247 Republic of Korea

**Keywords:** Axial spondyloarthritis, Radiographic progression, Artificial neural network, Quantitative prediction, Real-world setting

## Abstract

**Background:**

Predicting radiographic progression in axial spondyloarthritis (axSpA) remains limited because of the complex interaction between multiple associated factors and individual variability in real-world settings. Hence, we tested the feasibility of artificial neural network (ANN) models to predict radiographic progression in axSpA.

**Methods:**

In total, 555 patients with axSpA were split into training and testing datasets at a 3:1 ratio. A generalized linear model (GLM) and ANN models were fitted based on the baseline clinical characteristics and treatment-dependent variables for the modified Stoke Ankylosing Spondylitis Spine Score (mSASSS) of the radiographs at follow-up time points. The mSASSS prediction was evaluated, and explainable machine learning methods were used to provide insights into the model outcome or prediction.

**Results:**

The *R*^*2*^ values of the fitted models were in the range of 0.90–0.95 and ANN with an input of mSASSS as the number of each score performed better (root mean squared error (RMSE) = 2.83) than GLM or input of mSASSS as a total score (RMSE = 2.99–3.57). The ANN also effectively captured complex interactions among variables and their contributions to the transition of mSASSS over time in the fitted models. Structural changes constituting the mSASSS scoring systems were the most important contributing factors, and no detectable structural abnormalities at baseline were the most significant factors suppressing mSASSS change.

**Conclusions:**

Clinical and radiographic data-driven ANN allows precise mSASSS prediction in real-world settings. Correct evaluation and prediction of spinal structural changes could be beneficial for monitoring patients with axSpA and developing a treatment plan.

## Introduction

Axial spondyloarthritis (axSpA), including ankylosing spondylitis (AS), is a chronic progressive disease characterized by inflammation of the entheses, leading to new bone formation and ankylosis of joints, primarily in the axial skeleton [1, 2]. Radiographic progression of the spine has been reported to occur in approximately 20–50% of patients with AS after 2 years [3–5]. Progressive structural deformity of the spine and ankylosis of the sacroiliac joints lead to functional impairments, resulting in decreased physical activity and worsened quality of life.

Current treatment strategies have been validated to control the symptoms and disease activity of axSpA [6–8]. However, it remains inconclusive whether any currently available medications for axSpA have a significant effect on spinal radiographic progression [7]. Several factors predicting spinal radiographic progression have been identified, including male sex, smoking, presence of syndesmophytes at baseline, high degree of sacroiliitis on magnetic resonance imaging (MRI), and positivity for HLA-B27 [1, 3–5, 9–12]. Long-term use of tumor necrosis factor (TNF) inhibitors and effective suppression of inflammation also contribute suppressing the spinal radiographic progression in patients with AS [8, 11, 13, 14]. The modified Stoke Ankylosing Spondylitis Spinal Score (mSASSS) is a validated outcome measure for evaluating the effect of treatment on spinal radiographic progression in AS, and radiographs at 2-year intervals are usually required to ensure sufficient sensitivity to change [15]. These results were obtained from well-designed controlled trials and cohort studies. However, they had limitations in their application to individual patients in a real-world setting because the number of risk or protective factors differed across the patients, and their weights and interactions among them are complex and cannot be quantitatively measured in a formulated metric. Moreover, each patient’s visit schedule to the hospital varies according to lifestyle, work environment, and disease status. The time intervals of follow-up radiographs are also variable and not controlled for 2 years.

In previous studies, a novel subgroup of axSpA with a high risk for spinal radiographic progression was identified using machine learning (ML) algorithms and the ensemble method, and radiographic progression was predicted by a combination of clinical and radiographic variables [12, 16]. However, radiographic progression was defined as dichotomous discrimination [a change of ≥ 2 mSASSS units in 2 years (yes/no) or at least one new syndesmophyte formation in 2 years (yes/no)] that is qualitatively determined [12]. If radiographic progression could be precisely and quantitatively predicted, it would be more useful to monitor the disease course of patients and assess the treatment response. In this study, using a longitudinal observational cohort of patients with axSpA and linear regression and deep neural network models, we aimed to develop a fitted model to quantitatively predict the mSASSS at a specific follow-up time point with baseline clinical characteristics, radiographic damage indices, time-adjusted inflammatory burden, and exposure to treatment [non-steroidal anti-inflammatory drugs (NSAIDs) and TNF inhibitors].

## Methods

### Patients

A total of 682 patients with axSpA who fulfilled the Assessment of Spondyloarthritis International Society (ASAS) classification criteria for axSpA [17] and had received care at St. Vincent’s Hospital, Catholic University of Korea (Suwon, Republic of Korea), between 2005 and 2021 were identified. Clinical, laboratory data, and radiographic images were retrieved from medical records. At baseline, sex, age at diagnosis, time since diagnosis, HLA–B27 status, smoking status, and history of extra-articular manifestations (uveitis, psoriasis, inflammatory bowel disease, peripheral arthritis, and enthesitis) were recorded. Disease activity was assessed according to the ankylosing spondylitis disease activity score (ASDAS) using the C-reactive protein (CRP) level [18]. Dose and duration of NSAID intake, TNF inhibitor use, and treatment duration were determined. Records about the use of interleukin (IL)-17 inhibitor were excluded from this analysis because the number of patients treated with IL-17 inhibitors was too small to train the models. Of these, 555 patients underwent radiographic evaluation at more than two time points. Using the age- and sex-matched approach, the dataset was divided into training and testing datasets at a 3:1 ratio, and the training and testing datasets with the highest similarity in the follow-up time points and radiographic progression were selected from 1000 simulations. An ML model was learned on training data and validated on testing data. In total, 2034 follow-up radiographic time points were identified in 555 patients with axSpA. We filtered the follow-up radiographic time points over 12 months, and 1297 and 420 follow-up radiographic time point were identified in the training and testing datasets, respectively. The study was conducted in accordance with the Helsinki Declaration and was approved by the Institutional Review Board of St. Vincent’s Hospital, The Catholic University of Korea (No. VC22RISI0237).

### Radiographs and scoring

Radiographs of the sacroiliac joints and the cervical and lumbar spine were obtained at baseline and after follow-up. All available radiographs per patient were independently scored simultaneously according to the mSASSS [19] by two experienced readers, blinded to all other data except radiograph chronology. The interobserver reliability was assessed by calculating the interclass correlation coefficient, which was 0.946 (95% confidence interval [CI] 0.940–0.952). If the difference between the scores measured by the two readers was > 5 units (defined as major disagreement), the same assessors rescored these radiographs. In case of persistent major disagreement after rescoring, an independent adjudicator assigned a final score. Radiographic sacroiliitis (SI) was scored according to the modified New York criteria [20], and radiological hip involvement was graded based on the Bath Ankylosing Spondylitis Radiology Index (BASRI)-hip scoring system [21].

### Calculation of NSAIDs intake and exposure to TNF inhibitors

Data on NSAID intake (dose and frequency) were retrieved from medical records. An index of NSAID intake, as recommended by Assessment of SpondyloArthritis International Society (ASAS), accounting for both dose and duration/regimen of drug intake (0: no NSAIDs intake at all; 100: daily NSAIDs intake at a dose equivalent to diclofenac 150 mg over the whole period of interest) was calculated [22]. Exposure to TNF inhibitors was indicated as 0 if the patient did not receive anti-TNF therapy and as duration (months) if the patient was treated with TNF inhibitors.

### Calculation of time-integrated CRP levels

The inflammatory burden over the disease course was estimated using time-integrated CRP, calculated by the area under the curve method [23].

### Supervised ML algorithms for regression

**Fig. 1:**
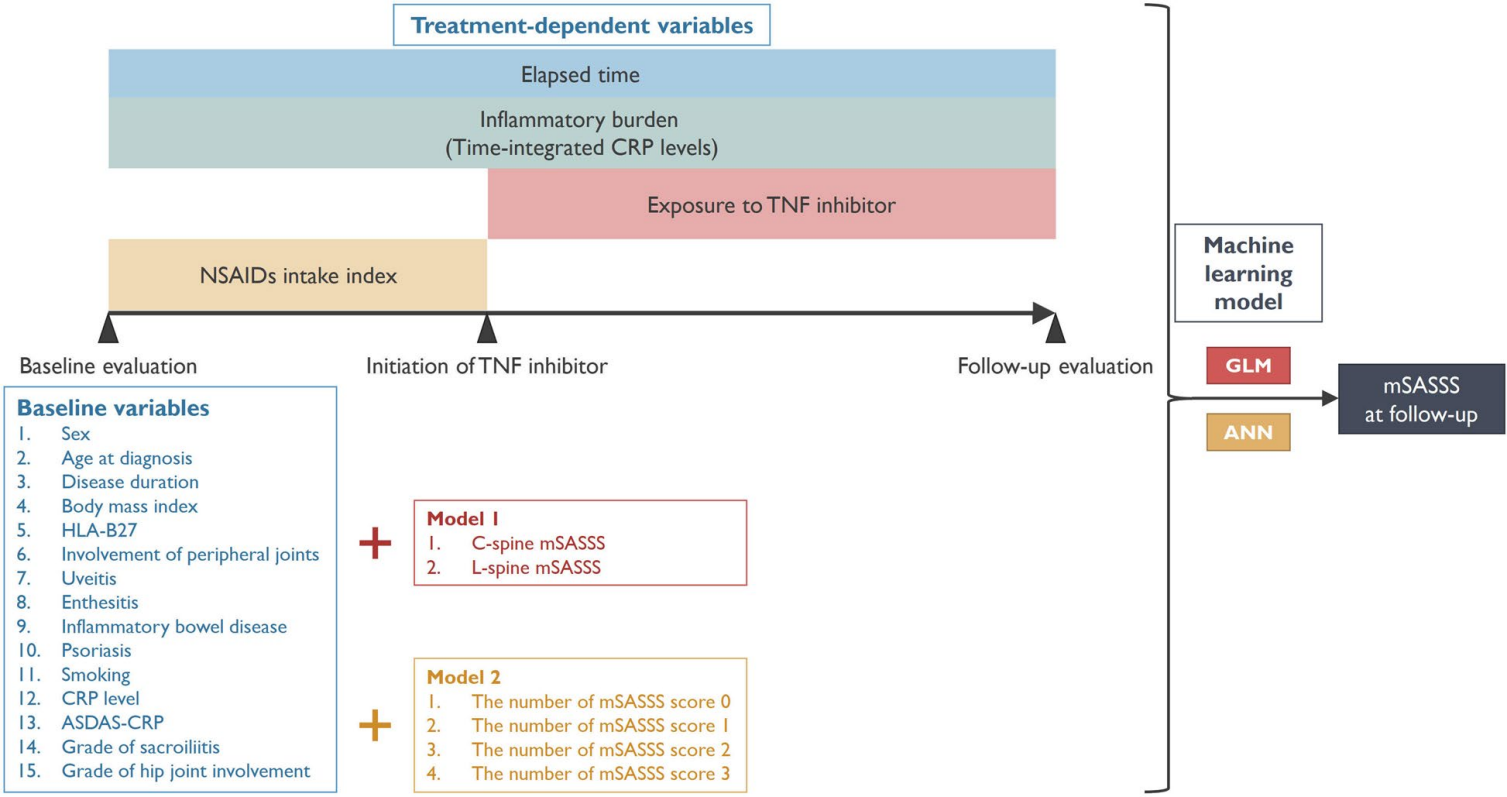
Overview of the development of the mSASSS prediction model. The known and potential factors affecting the radiographic progression were included in the formulation of the GLM or ANN model (blue box). Treatment-dependent variables include the elapsed time after baseline evaluation, time-integrated CRP levels, and exposure to TNF inhibitors. Baseline mSASSS was modified into two formats and assigned to the models (red and yellow box): (1) C-spine and L-spine mSASSS and (2) number of each score of mSASSS (0, 1, 2, and 3). Target outcome was the mSASSS at follow-up. Finally, two models were built by the formats of mSASSS and separately evaluated. ANN, artificial neural network; ASDAS, Ankylosing Spondylitis Disease Activity Score; CRP, c-reactive protein; C-spine, cervical spine; GLM, generalized linear model; L-spine, lumbar spine; mSASSS, modified Stoke Ankylosing Spondylitis Spinal Score; NSAID, non-steroidal anti-inflammatory drug; TNF, tumor necrosis factor

The scheme of the supervised ML is illustrated in Fig. 1. Two ML models were applied to predict the mSASSS at a specific follow-up time point: a generalized linear model (GLM) [24] and artificial neural network (ANN) model [25, 26]. GLM is the simplest ML algorithm for specifying the relationship between a weighted sum of the feature inputs and a single numeric target. An ANN consists of units arranged in layers to convert an input vector into an output. The layers between the input and output layers are often hidden. Each unit receives an input, applies a function, and passes it to the next layer. Weights were applied to the signals passing from one unit to another, which were modified during the training phase. Backpropagation allows the model to self-learn [26]. A multi-layered ANN with a backpropagation algorithm was trained, a total of 1000 iterations of the ANN with three, five, seven, or nine hidden layers were simulated, and the best model with the highest performance was selected. We used the neuralnet function installed in the R package neuralnet as the default settings [27, 28]. Neuralnet function uses a globally convergent algorithm (grprop) based on resilient backpropagation without weight backtracking and additionally modifies one learning rate. The logistic function (*f*(*u*) = 1/(1 + *e*^−*u*^)), a bounded nondecreasing nonlinear and differentiable function, was used as an activation function, and the learning rates in the grprop algorithm are limited to the boundaries from the lower 0.5 to the upper 1.2 [29].

### Explainable ML model interpretation

Two methods were used to interpret the model: (1) variable importance measured by the model-agnostic method [30] and (2) Shapley additive explanations (SHAP). In the model-agnostic method, if a variable is important, then we expect that the model’s performance will worsen after permuting the variable’s values. The significance of the variable increases with the extent of the performance variation. SHAP explains any model’s prediction by computing each feature’s contribution to the prediction [31, 32]. This method is based on Shapley values from coalitional game theory, which is the average marginal contribution across all possible coalitions [33]. The SHAP value of a clinical variable *V* (e.g., NSAIDs intake index) is computed as the average of this variable’s contributions across all possible combinations of clinical variables, including *V*. The SHAP value of a clinical variable can be positive or negative, suggesting an increased or decreased likelihood of developing a particular outcome [32]. Our study investigated the impact and interaction among clinical variables by visualizing SHAP values in global (cohort level) forms.

### Evaluation of predictive performance for the regression model

Three error metrics were used to evaluate the performance of the regression model: (1) mean squared error (MSE), (2) root mean squared error (RMSE), (3) mean absolute error (MAE), and the coefficient of determination (*R*^2^) [34]. The MSE of an estimator measures the average squared difference between estimated and true values. The RMSE is a rooted, monotonic transformation of the MSE. The MAE measures the average of the sum of the absolute differences between the observed and predicted values. The coefficient of determination is the proportion of variation in the dependent variable that is predictable from independent variables.

### Statistical analyses

For continuously distributed data, the results are shown as means with standard deviation; between-group comparisons were performed using Student’s *t*-test or analysis of variance (ANOVA). Categorical or dichotomous variables were presented as frequencies and percentages and were compared using the chi-squared test or Fisher’s exact test. Correlation analysis between two continuous variables was performed using Pearson’s method. A two-sided *P*-value less than 0.05 was considered statistically significant. All statistical analyses were performed using R (version 4.2.0, R Project for Statistical Computing, www.r-project.org).

## Results

### Baseline characteristics of the study population

**Fig. 2:**
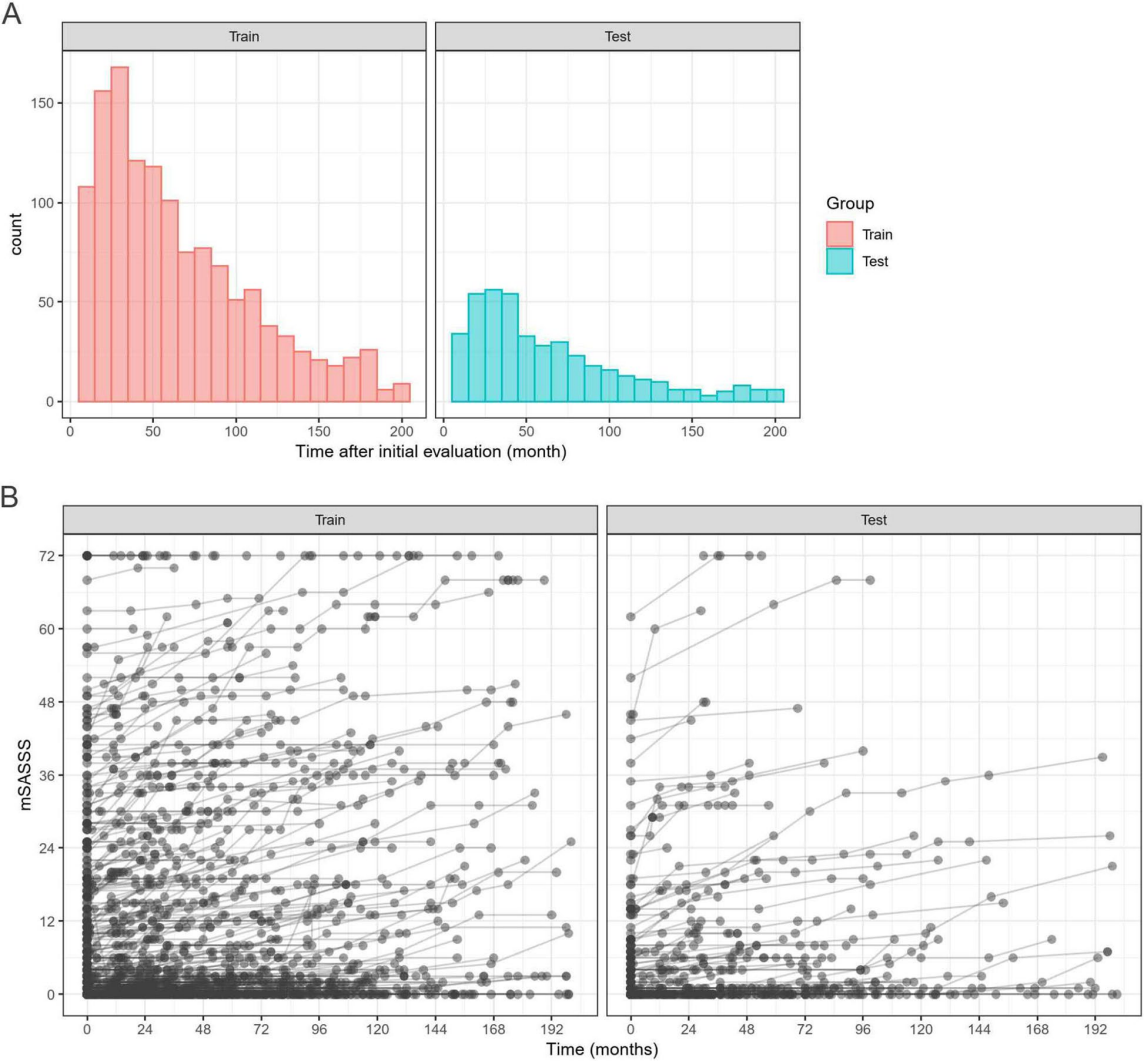
**A** Follow-up time points in the training and testing datasets. **B** Sequential change in mSASSS of the individual patients by follow-up time points in the training and testing datasets

In total, 555 patients with axSpA were enrolled and split into training (*n* = 416, 75%) and testing (*n* = 139, 25%) groups in an age- and sex-matched stratified manner. The baseline characteristics of the study participants (*n* = 555) are presented in Table 1. All baseline characteristics, except for a history of enthesitis were comparable between the groups. In total, 310 patients with axSpA (55.8%) received TNF inhibitors. The number of follow-up time points in the training and testing datasets was 1297 and 420, respectively (Fig. 2).

**Table 1:** Baseline characteristics: training versus testing groups

Variable	Training (*n* = 416)	Testing (*n* = 139)	*P* value^b^
Male, *n* (%)	310 (74.5)	107 (77.0)	0.640
Age, years	43.7 ± 13.0	43.3 ± 13.2	0.721
BMI, kg/m^2^	24.1 ± 4.0	24.0 ± 3.8	0.875
HLA-B27, *n* (%)	336 (80.8%)	109 (78.4)	0.833
Smoking			0.640
Never, *n* (%)	231 (55.5)	73 (52.5)	
Ex-smoker, *n* (%)	49 (11.8)	13 (9.4)	
Current smoker, *n* (%)	136 (32.7)	53 (38.1)	
Peripheral arthritis, *n* (%)	120 (28.8)	40 (28.8)	0.223
Enthesitis, *n* (%)	35 (8.4)	21 (15.1)	0.035
Uveitis, *n* (%)	109 (26.2)	29 (20.9)	0.251
Psoriasis, *n* (%)	20 (4.8)	9 (6.5)	0.586
Inflammatory bowel disease, *n* (%)	13 (3.1)	3 (2.2)	0.766
ESR, mm/h	38.2 ± 29.4	36.1 ± 28.3	0.462
CRP, mg/dL	2.0 ± 3.3	1.7 ± 2.7	0.223
ASDAS-CRP	3.4 ± 0.9	3.3 ± 0.9	0.836
Use of TNF inhibitor, *n* (%) ^a^	233 (56.0)	77 (55.4)	0.978
Presence of syndesmophyte(s), *n* (%)	161 (38.7)	100 (38.9)	1.000
mSASSS, units	8.5 ± 14.5	6.6 ± 12.6	0.187
Cervical spine	3.3 ± 6.9	2.5 ± 6.1	0.269
Lumbar spine	5.2 ± 9.1	4.1 ± 7.6	0.166

*Abbreviations*: *ASDAS* Ankylosing Spondylitis Disease Activity Score, *BMI* Body mass index, *CRP* c-reactive protein, *ESR* Erythrocyte sedimentation rate, *mSASSS* modified Stoke Ankylosing Spondylitis Spine Score, *TNF* Tumor necrosis factor

^a^TNF inhibitors include etanercept, adalimumab, infliximab, and golimumab

^b^Intergroup comparisons were performed using the Student’s *t*-test for continuously distributed data and the chi-squared test for categorical variables

### Linear regression models for mSASSS prediction

Known and potential factors affecting radiographic progression were included while the formulation of the linear regression model: sex, age at diagnosis, disease duration, body mass index (BMI), HLA-B27, peripheral involvement, uveitis, enthesitis, inflammatory bowel disease, psoriasis, smoking, baseline CRP level, baseline ASDAS-CRP, grade of sacroiliitis, grade of hip joint involvement, and baseline mSASSS. Treatment-dependent variables included time after baseline evaluation, time-integrated CRP level, and exposure to TNF inhibitors. If the patient did not receive a TNF inhibitor, exposure to the TNF inhibitor was assigned to zero. Baseline mSASSS was modified into two formats and assigned to the models: (1) C-spine and L-spine mSASSS and (2) the number of each score of mSASSS scores (0, 1, 2, and 3). Finally, two GLM models (designated as GLM-1 and GLM-2) were built using the formats of mSASSS and separately evaluated (Fig. 1).

**Fig. 3:**
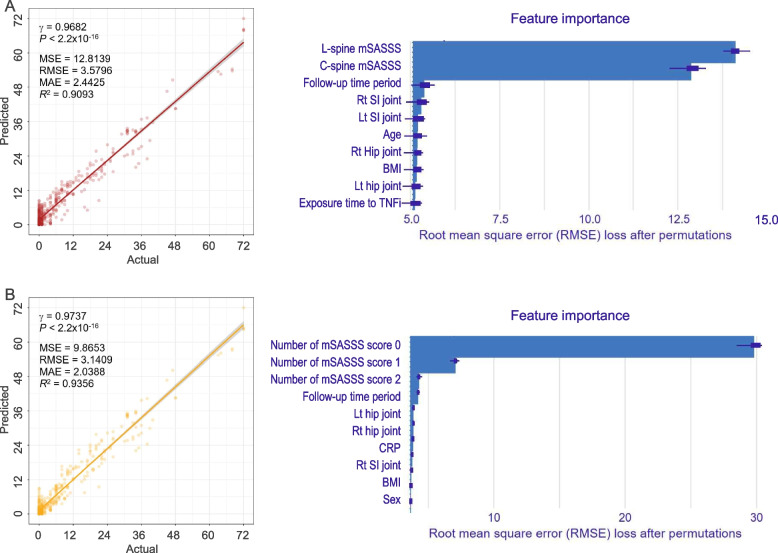
Linear regression models for mSASSS prediction. **A** GLM-1 with baseline mSASSS as a total score. **B** GLM-2 with baseline mSASSS as the number of each score. Scatterplots of actual versus predicted mSASSS (left panel) and bar plot of feature importance (right panel). GLM, generalized linear model; MAE, mean absolute error; MSE, mean squared error; RMSE, root mean squared error

The prediction results of mSASSS in the testing dataset are shown in Fig. 3. For GLM-1, *R*^2^ and RMSE values were 0.9093 and 3.5796, respectively. The most important variables for prediction were baseline mSASSS of the L-spine and C-spine, followed by the time after the initial evaluation. For GLM-2, *R*^2^ and RMSE values were 0.9356 and 3.1409, respectively. The number of mSASSS segment scores 0, 1, and 2 were counted as important variables, but the number of mSASSS segment scores 3 was not. The time after the initial evaluation was also an important variable.

### ANN models for mSASSS prediction

**Fig. 4:**
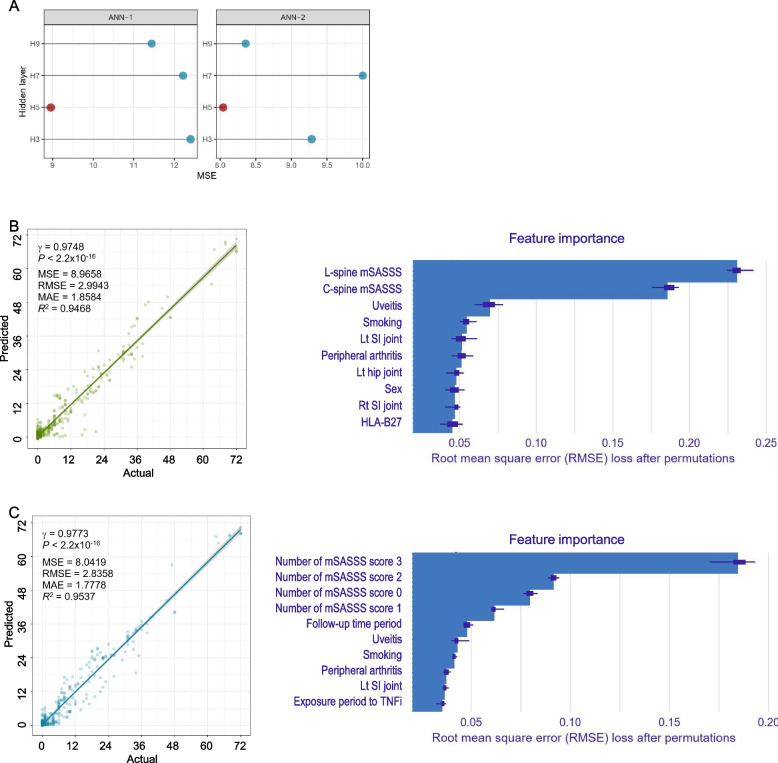
Artificial neural network model for mSASSS prediction. **A** MSE by the number of hidden layers. **B** ANN-1 with baseline mSASSS as a total score. **C** ANN-2 with baseline mSASSS as the number of each score. Scatterplots of actual versus predicted mSASSS (left panel) and bar plot of feature importance (right panel). ANN, artificial neural network; MAE, mean absolute error; MSE, mean squared error; RMSE, root mean squared error

Same as that in the GLM, the baseline mSASSS was modified into two formats, and two ANN models (designated as ANN-1 and ANN-2, respectively) were built using mSASSS format. A multi-layered ANN with a backpropagation algorithm and three, five, seven, or nine hidden layers was fitted, and the best model with the highest performance was selected. In both ANN-1 and ANN-2, models of five hidden layers showed the best performance compared to models of three, seven, or nine layers by MSE (Fig. 4A).

For ANN-1 with five hidden layers, the *R*^2^ and RMSE values were 0.9468 and 2.9943, respectively (Fig. 4B). The most important two variables for prediction were the same for GLM-1 (baseline mSASSS of the L-spine and C-spine), but ANN-1 showed better performance than GLM-1. The third most important variable was the time after the initial evaluation in GLM-1, while positive history of uveitis in ANN-1.

For ANN-2, with five hidden layers, the *R*^2^ and RMSE values were 0.9537 and 2.8358, respectively (Fig. 4C). This model showed the best performance. The number of mSASSS segment scores of 3 and 2 were considered the most important variables, followed by the number of mSASSS segment scores of 0 and 1. Time after the initial evaluation, history of uveitis, and smoking status were also important variables. Exposure time to TNF inhibitors was identified as having some contribution to ANN-2.

**Fig. 5:**
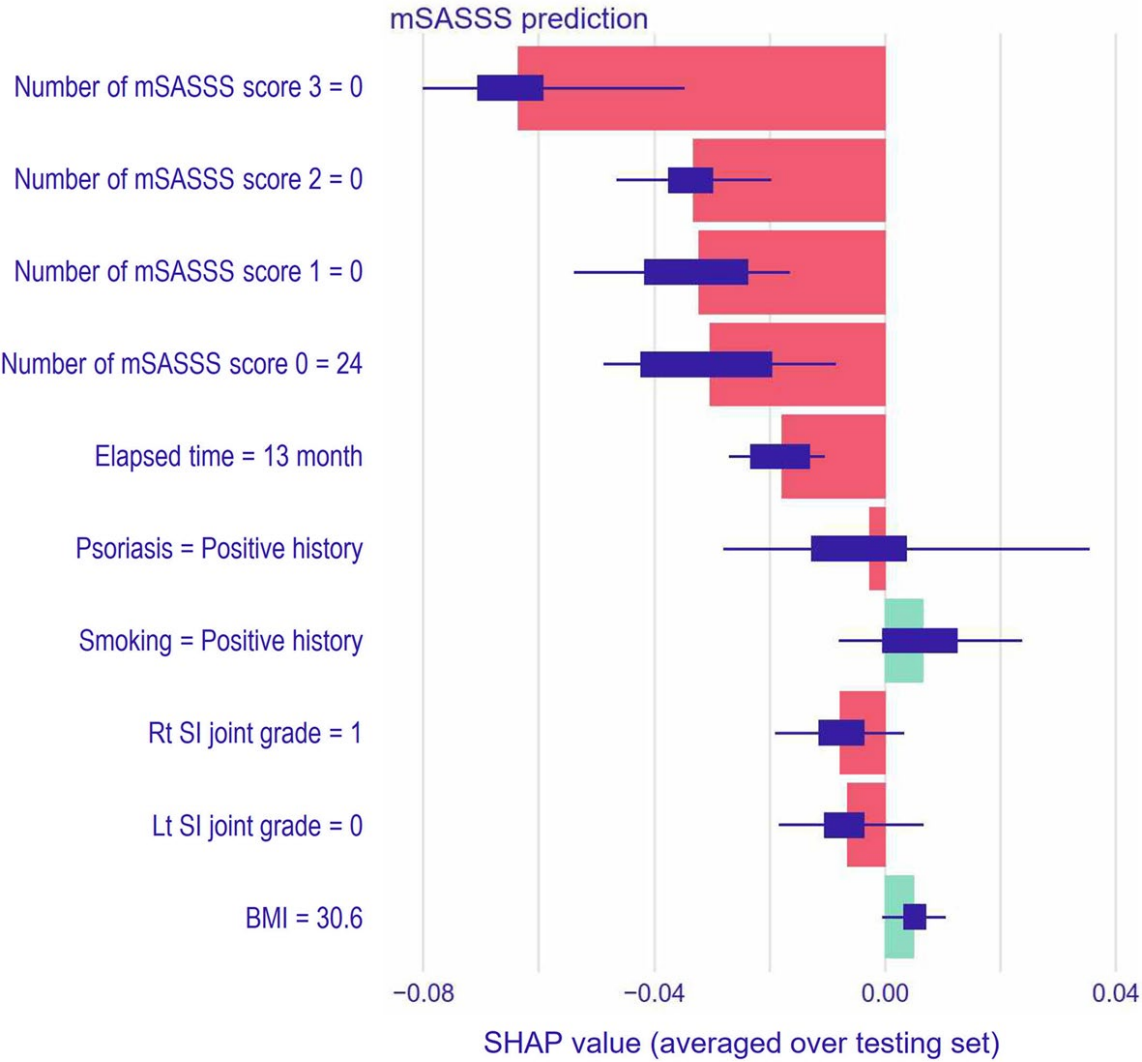
A bar plot of the average Shapley additive explanation (SHAP) value for each predictor

Figure 5 shows the SHAP summary plot for the top 10 features contributing to the ANN-2 model’s prediction of follow-up mSASSS in patients with axSpA. No 3 and 2 scores in the mSASSS segments (i.e., no bridged syndesmophytes) and zero scores for all 24 segments in the mSASSS (i.e., total mSASSS = 0) at baseline evaluation exercised strong leverage on mSASSS change in a negative way. Short-term follow-up (indicated as 13 months after the initial evaluation in this analysis) also had a negative effect on increase in mSASSS prediction. Smoking and being overweight (indicated as BMI = 30.6 kg/m^2^) contributed to increase in mSASSS prediction at follow-up. Overall, contribution of minor factors was distinctly sensed in the ANN compared to the GLM.

When subdivided into three subgroups by follow-up time points (less than 2 years, 2–4 years, and over 4 years), the RMSE tended to decrease as the follow-up time increased (Table 2). The RMSE was much smaller in patients without syndesmophytes at baseline than in those with syndesmophytes at baseline (Table 2).

**Table 2:** Subgroup analysis of performance in mSASSS prediction

Category	Subgroup	*n*	MSE	RMSE	MAE
Elapsed time	< 2 years	81	9.47	3.08	1.68
	2–4 years	125	8.15	2.85	1.78
	> 4 years	214	7.44	2.73	1.81
Syndesmophyte(s)	Absence	280	2.35	1.53	0.976
	Presence	140	19.40	4.41	3.38

*MSE* Mean squared error, *RMSE* Root mean squared error, *MAE* Mean absolute error, *mSASSS* Modified Stoke Ankylosing Spondylitis Spine Score

## Discussion

In this study, we demonstrated the feasibility of ML models in predicting mSASSS using baseline clinical characteristics and treatment-dependent variables, which were obtained in clinical practice but were quite diverse. The mSASSS was predictable beyond the limit of the simplified binary definition of radiographic progression in 2 years. The performance was excellent in that the *R*^2^ values of the fitted models were in the range of 0.93–0.96. In particular, ANN performed better than GLM and effectively captured the complex interactions among variables and their contributions to the transition of mSASSS over time in the fitted models.

In our analysis, the input of mSASSS as a format of the number of each score had a better predictive power compared to the input of mSASSS as a format of the total score, indicating that fractionized scoring data is more suitable for building the mSASSS prediction model than the summed-up single value. Radiographic damage in the axSpA linearly progresses at a variable rate and is scored in the range of 0–72 by mSASSS. Each score (0, 1, 2, and 3) represents its own structural abnormalities and a distinct pathophysiological background. Even the same total score could indicate different structural damages. Each lesion could also respond differently to treatment according to the adjacent internal tissue status, such as fat deposition or metaplasia on MRI [35, 36]. The total score of the mSASSS and dichotomous definition of radiographic progression is useful for easy recognition and prompt assessment of spinal structural damage in clinical practice. However, it might be too simplified to present the substantive condition. Categorizing continuous variables by an arbitrary cutoff point can lead to the loss of important information or overestimation or underestimation [37]. The presence of syndesmophyte(s) at baseline was a powerful established predictor for radiographic progression within 2 years [1, 38]. However, total mSASSS was a more important feature for predicting radiographic progression than the presence of syndesmophyte(s) in most ML algorithms [12], which effectively deal with high-dimensional complex data, including multiple heterogeneous factors contributing to the disease [39, 40]. More detailed and fragmented data could be more informative for making a predictive model with better performance in ML processing.

In ANN, models with five hidden layers showed the best performance compared to models of three, seven, or nine layers. This indicates that deeper ANN did not necessarily demonstrate better performance. Simple algorithms can perform just as well as or even better than more complex ones in some circumstances: when the underlying relationship between features and output is simple and additive or when the number of training examples is relatively low. Thus, more complex models are likely to overfit and generalize poorly [41]. Clinical data are not as highly complex as radiographic images, magnetic resonance images, or multi-omics data and might not fit the deeper or sophisticated ANN [39, 40]. In the subgroup analysis, the mSASSS prediction was more accurate with a longer follow-up period or in the absence of syndesmophytes at baseline. The short-term follow-up data may not have been sufficiently learned because there were relatively few data points (Fig. 2A); moreover, the complexity of the interaction between variables could be lower in the long-term stable stage. Syndesmophytes result from new bone formation that develops after initiating an inflammatory event [42, 43]. The presence of syndesmophyte(s) indicates that the bone-forming potential might exceed the control threshold of inflammation. In our analysis, laboratory data and treatment strategies largely depended on the inflammatory process (e.g., NSAID intake index, exposure to TNF inhibitor, and time-integrated CRP levels) and did not include any specific information regarding bone formation such as bone formation biomarkers and sequential MRI findings. Thus, the decreased accuracy of mSASSS prediction in the presence of syndesmophyte(s) might be attributable to insufficient information.

ANN showed better performance and better discerned the complex interaction among variables and their contribution to the outcome compared to GLM. Radiographic progression is structural damage as an aggregated result of interactions between clinical, molecular, and environmental factors and cannot be fully explained by simple and additive models. ANN traditionally had a concern, so-called black-box problem. The problem-solving process in artificial intelligence is opaque and not interpretable to humans in a straightforward manner. Feature importance and SHAP analyses are solutions in the field of explainable ML and are used to gain insight into model performance and the contribution of various risk factors. Structural changes constituting the mSASSS scoring systems were the most important contributing factors, and no detectable structural abnormalities at baseline were the most significant factors suppressing the mSASSS change. This finding corroborates the importance of early diagnosis and initiation of effective treatment before spinal structural changes begin.

This study had some limitations. First, the data were retrospectively collected. Retrospective data collection is susceptible to misclassification and information bias. Second, this study lacked bone formation markers or MRI findings, which could be informative for new bone formation in axSpA. Third, mSASSS has inherent limitations: the inability to assess involvement of the thoracic spine and facet joints, which are the most frequently affected sites of axSpA [15].

## Conclusions

In conclusion, interventions that slow or halt the progression of irreversible structural damage in axSpA are expected to confer clinical benefits in terms of delaying loss of function and improving the quality of life. Correct estimation of the disease and prediction of treatment response should be beneficial for evaluating the treatment response and making a future plan. Our study showed that the constructing predictive models for radiographic progression were feasible in a real-world setting and that the models displayed good performance. Prospective studies examining the use of ML in mSASSS prediction in a multicenter cohort with a larger size are needed to validate the use of such models. The discovery of clinically active biomarker(s) in terms of new bone formation and the development of exact assessment tools could also boost the development of a better predictive model for radiographic progression in axSpA.

## Data Availability

The data underlying this article cannot be shared publicly for the protection of the privacy of individuals that participated in the study. The data may be shared upon reasonable request to the corresponding author.

## Abbreviations

ANN: Artificial neural networks
ANOVA: Analysis of variance
AS: Ankylosing spondylitis
ASAS: Assessment of Spondyloarthritis International Society
ASDA: Ankylosing spondylitis disease activity score
axSpA: Axial spondyloarthritis
BASRI: Bath Ankylosing Spondylitis Radiology Index
BMI: Body mass index
CRP: C-reactive protein
GLM: Generalized linear model
IL: Interleukin
MAE: Mean absolute error
ML: Machine learning
MRI: Magnetic resonance imaging
mSASSS: Modified Stoke Ankylosing Spondylitis Spinal Score
MSE: Mean squared error
NSAIDs: Non-steroidal anti-inflammatory drugs
R2: The coefficient of determination
RMSE: Root mean squared error
SHAP: Shapley additive explanations
SI: Sacroiliac
TNF: Tumor necrosis factor
